# Evaluation of Global Differential Gene and Protein Expression in Primary Pterygium: S100A8 and S100A9 as Possible Drivers of a Signaling Network

**DOI:** 10.1371/journal.pone.0097402

**Published:** 2014-05-13

**Authors:** Aihua Hou, Wanwen Lan, Kai Pong Law, Ser Chin Jasmine Khoo, Min Qi Tin, Yoon Pin Lim, Louis Tong

**Affiliations:** 1 Ocular Surface Research Group, Singapore Eye Research Institute, Singapore, Singapore; 2 Department of Biochemistry, National University of Singapore, Singapore, Singapore; 3 Duke-NUS Graduate Medical School, Singapore, Singapore, Singapore; 4 Singapore National Eye Center, Singapore, Singapore; 5 Yong Loo Lin School of Medicine, National University of Singapore, Singapore, Singapore; University of North Carolina School of Medicine, United States of America

## Abstract

**Purpose:**

Pterygium is a wing shaped fibrovascular growth on the ocular surface, characterized by fibrosis, angiogenesis, extracellular matrix remodeling, and inflammatory infiltrates. Epidemiologic studies have linked pterygium formation to various chronic inflammatory conditions, such as ultraviolet radiation, sawdust exposure, and dry eye disease. The purpose of this study is to identify proteins that are differentially expressed in primary pterygium by using a combination of gene microarray and proteomic platforms.

**Methods:**

Paired pterygium and uninvolved conjunctiva tissues of four patients were evaluated for differences in global gene transcript levels using a genechip microarray. Proteins extracted from another four pairs of tissues were quantified by iTRAQ approach. Western blot and immunofluorescent staining on additional patients were used to validate dysregulated protein expression obtained from microarray and proteomics data. In addition, primary conjunctival fibroblasts were treated with recombinant S100A8, S100A9 or both. Transcript level changes of a panel of potential target genes were evaluated by real time-PCR.

**Results:**

The following were up-regulated at both protein and transcript levels S100 A8 and A9, aldehyde dehydrogenase 3 family, member1 (ALDH3A1) and vimentin (VIM). Conversely, serpin peptidase inhibitor clade A member 1 (SERPINA1) and transferrin (TF) were down-regulated. Upon adding S100A8, S100A9 or both, the inflammatory chemokine CXCL1, matrix proteins vimentin, biglycan, and gelsolin, as well as annexin-A2, thymosin-β4, chymase (CMA1), member of Ras oncogene family RAB10 and SERPINA1 were found to be up-regulated.

**Conclusions:**

We identified 3 up-regulated and 2 down-regulated proteins by using a stringent approach comparing microarray and proteomic data. On stimulating cells with S100A8/9, a repertoire of key genes found to be up-regulated in pterygium tissue, were induced in these cells. S100A8/9 may be an upstream trigger for inflammation and other disease pathways in pterygium.

## Introduction

Pterygium is an ocular surface disease which characterized by abnormal epithelial and fibrovascular proliferation, invasion, and matrix remodeling. The lesion leads to astigmatism, ocular irritation, dryness, and reduced vision once it involves the visual axis. Despite its prevalence, the exact pathogenesis of pterygium remains unknown. Evidence from epidemiology and histopathology studies support the concept that pterygium arises as a result of ultraviolet (UV) radiation-damage [1]–[4]. UV radiation induces damage in DNA and other cellular substrates [5]–[7]. In addition, several studies have pointed to an immune response-inflammatory component in pterygium pathology [8]–[11].

Previously, microarray techniques have been used to compare the genome wide gene expression profiles between primary pterygium and normal conjunctiva tissues [12]–[14]. Extracellular-matrix-related, proinflammatory, angiogenic, fibrogenic, and oncogenic genes were found to be dysregulated in primary pterygium [13]. About 1724 genes were significantly different between primary and recurrent pterygium by genome-wide microarray scanning [15]. Our group reported 51 genes with at least 2 fold changes between primary and recurrent pterygium [14]. More recently, proteomic techniques have been applied in pterygium research. Using two-dimensional electrophoresis and mass spectrometry (2D-MS), Kanamoto *et al*. identified 11 differentially expressed proteins between primary and recurrent pterygium [16]. Using a similar assay technique, peroxiredoxin 2 was found to be detected in primary pterygium tissue [17].

The S100A8 and S100A9 are calcium binding secreted proteins that have been previously identified to be up-regulated in various ocular surface conditions including pterygium, dry eye, meibomian gland dysfunction and corneal neovascularisation [18]–[21]. In living tissues, these two proteins may exist as homodimers or heterodimers and play a big role in regulation of oxidative damage [22]–[28] as well as initiation of immune responses [24], [29]–[32]. Because of their possible role as a master regulator of immune response and inflammation, S100A8/S100A9 deserve further study. The relationship of S100A8 and S100A9 and downstream inflammatory molecules has not been evaluated in the context of the ocular surface.

Since there are advantages to an unbiased molecular profiling method in a disease with uncertain etiology, we aimed to identify dysregulated molecules in primary pterygium tissue compared to normal uninvolved conjunctiva by proteomics evaluation and comparing findings with gene microarray analysis. We further investigated the ability of S100A8, S100A9 and S100A8/A9 to regulate the expression of a panel of genes involved in pterygium pathology using primary cell cultures.

## Methods and Materials

### 1. Patients and Ethics Statement

Primary nasal pterygium and uninvolved conjunctival tissues from the superior temporal quadrant of the same eye were collected and stored immediately in RNA*later* RNA Stabilization Reagent (Qiagen) for RNA extraction, or immediately frozen in liquid nitrogen for protein extraction. All procedures adhered to the tenets of the Declaration of Helsinki and were approved by the Institutional Review Board of the Singapore Eye Research Institute. Written informed consent was obtained from all participating patients.

### 2. Microarray Gene Expression Evaluation

RNA was extracted from 4 pairs of tissues using the following protocol: Tissues were minced into small pieces, and ground into powder using pestle and mortar with liquid nitrogen. The tissue powder was transferred into TRIzol (Invitrogen) and passed through 22G needles several times (Becton Dickenson). Chloroform was added to each homogenized sample, mixed well and centrifuged at 13,000 rpm for 15 min at 4°C. The aqueous layer was transferred to a new tube and an equal volume of 70% ethanol added. Subsequently the samples were purified using RNeasy RNA extraction kit (Qiagen) according to manufacturer’s protocol. RNA concentration and quality were assessed using the ND-1000 spectrophotometer (Thermo Scientific).

The microarray experiment was performed on the Agilent Sureprint G3 human GE 8×60 K (ID: G6851-60510) single-colour chip by a commercial service (Molecular Genomics Pte Ltd, Singapore) according to the Agilent protocol. In summary, one-hundred nanograms of total RNA was labeled with Low Input Quick Amp Labeling Kit (One-Colour Microarray), then converted to double-stranded cDNA using oligo-dT primers and transcribed by T7 RNA polymerase to produce cyanine 3-CTP labeled cRNA. Sixty nanograms of labeled cRNA was hybridized onto Agilent SurePrint G3 human GE 8×60 K microarray for 17 hrs at 65°C, 10 rpm in Agilent hybridization oven. After hybridization, the microarray slide was washed and scanned on Agilent High Resolution Microarray scanner. Raw signal data was extracted with Agilent Feature Extraction Software (V10.7.1.1), and analyzed by GeneSpring 12.5 (Agilent, CA).

The threshold of raw signals was set to 1.0 and data was transformed to logbase 2 values. Per chip normalization, performed by baseline transformation to the median of all samples, was performed by percentile shift to 75^th^ percentile. A box plot of normalized data for the 8 samples showed a similar distribution. Subsequent quality control(QC) on the probeset to remove faulty or suspicious probes was done by filtering on flags (probes flagged as ‘not detected’ and ‘compromised’) and filtering on expression (removal of probes with very low raw signal intensity (<20)). This QC processing identified 27714 positive signals. The microarray data reported in this study had been deposited in NCBI Gene Expression Omnibus (GEO, http://www.ncbi.nlm.nih.gov/geo) with GEO series record number GSE51995.

### 3. ITRAQ-LC-MS/MS Proteomics

#### Protein extraction and quantification

Four pairs of tissues were weighed and washed with phosphate buffered saline (PBS), and then put into 100 µl of lysis buffer (75 mM NaCl, 1 mM EDTA, 0.2% CHAPS, 50 mM Sodium Fluoride, 1 mM sodium orthovanadate and 1× protease inhibitors cocktail prepared in PBS). Ten freeze-thaw cycles involving snap-freezing with liquid nitrogen and thawing on ice water were subsequently performed. Samples were then ground using a mini grinder, and 400 µl of the lysis buffer was added. Ground samples were further homogenized via sonication. Triton-X (0.2%) and IGEPAL (0.2%) were added after sonication. Homogenized samples were spun at 16,000 g for 20 minutes at 4°C, the supernatant was concentrated to approximately 50 µL by a 0.5 ml 3 KDa ultra-centrifugal filter (Amicon, USA) and the protein concentration were measured by Bradford assay.

#### Mass spectrometry and data analysis

Samples were reduced, alkylated, digested and labeled with iTRAQ following the manufacturer’s instruction. The combined samples were cleaned and fractionated with strong cation exchange (SCX) chromatography on a PolySULFOETHYL A column (200×4.6-mm, 5 µm, 200 Å; PolyLC Inc., Columbia, MD, USA). Forty-one fractions were collected and they were subsequently pooled to 15 samples for mass spectrometry analysis. The collected fractions were first reduced volume and reconstituted in 1% formic acid/5% acetonitrile for desalting by solid phase extraction. C18 purification was performed on Pierce C18 Spin Columns (Thermo Scientific). The desalted samples were lyophilized and reconstituted in 20 µl of 0.1% formic acid/2% acetonitrile. LC-MS/MS analysis was performed on a QStar Elite quadruple time of flight mass spectrometer system (AB Sciex) coupled to a Tempo nano-LC (AB Sciex) with autosampler.

Database search and relative analysis was performed on ProteinPilot™ software (AB Sciex) version 4.0.8085, rev 148085 with the Paragon Algorithm™ version 4.0.0.0, rev 148083 using UniProtKB/Swiss-Prot database release 2013_12 (containing 49,243,530 entries) against the human genome (20274 sequences) [33]–[34]. Database search was performed by setting cysteine modification by MMTS as a fixed modification [35] and tryspin as the digesting enzyme. None of the special factors was selected. The unused Protscore (conf) in the software was set to 1.3 (95% confidence). Protein level changes were compared between paired samples of pterygium and conjunctiva, and iTRAQ fold change of >1.3 was considered to be up-regulated in pterygium, while a ratio of <0.77 was considered down-regulated. This is based on fact that the systematic variation of our analytical system has been determined consistently to be within 30% across several studies [36]–[41].

### 4. Primary Pterygium and Conjunctival Fibroblast Cell Culture

Primary pterygium and conjunctiva fibroblasts were cultured as previously reported [42]–[43]. Briefly, pterygium and conjunctiva tissues were immediately submerged in 1 ml of DMEM/F12 (Gibco) medium containing 1× antibiotic-antimycotic (1× A/A) (Invitrogen, USA) and transferred to lab on ice. Culture plates (60 mm) were coated with 1 ml of fetal bovine serum (FBS) (Gibco) for 10 min at 37°C. Each specimen was cut into pieces of approximately 1–2 mm^2^ and placed onto the FBS-coated plates. Sixty microliters of FBS with 1× A/A was dropped over each piece. Explants were incubated overnight at 37°C with 5% CO_2_. The next morning, 120 µl of DMEM/F12 with 10% FBS and 1× A/A were dropped over each explant, and 400 µl more of the same medium was added later in the evening. About 3–5 days later, cells started to migrate from explants and the volume of medium was increased to 2 ml and changed every other day. Fibroblasts were then sub-cultured at day 18∼21 using TrypL Express (Life Technologies). Cells from passage 1 to passage 3 were used for experiments. Cell population homogeneity was observed as previously described [44].

### 5. Western Blot

Western blots were performed as described [44]. In summary, total protein (30 µg) from primary cultured pterygium and conjunctiva fibroblast cells were electrophoresed in 10–12% SDS-polyacrylamide gels, and then transferred to polyvinylidene difluoride (PVDF) membranes at 4°C overnight. The membranes were subsequently incubated with primary antibodies to ALDH3A1 (Abcam, UK, Cat. No. AB76976, 1∶1000) and GAPDH (Abcam, UK, AB37168, 1∶1000) for 2 hrs at room temperature. Membranes were then incubated with secondary antibodies conjugated to horseradish peroxidase for 1 hr at room temperature. Immunoreactivity was visualized with chemiluminescence substrate (SuperSignal West Pico or West Dura; Pierce).

### 6. Immunofluorescent Staining

Pterygium and conjunctival tissues were sectioned with a Microm HM550 cryostat (Microm, Walldorf, Germany) at 8 µm thickness. Sections were fixed with 4% paraformaldehyde for 20 min, washed with PBS, permeated in PBS containing 0.15% Triton X-100 for 15 min, blocked with 4% BSA in PBS containing 0.1% Triton X-100 (Sigma) for 1 hr, and then incubated with ALDH3A1 antibody (Abcam, UK, Cat. No. AB76976, 1∶200 dilution in blocking solution) at 4°C overnight. After washing with PBS containing 0.1% Tween-20, the sections were incubated with Alexa Fluor 488-conjugated secondary antibody at room temperature for 45 min. Slides were then mounted with UltraCruz Mounting Medium (Santa Cruz). For negative controls, non-immune reactive serum was used in place of the specific primary antibody. Sections were observed and imaged using a Zeiss Axioplan 2 fluorescence microscope (Zeiss, Oberkochen, Germany).

### 7. S100A8, S100A9 and S100A8/A9 Treatment of Conjunctiva Fibroblast Cells

The maximum non-toxic dose of recombinant S100A8, S100A9 and combined S100A8/S100A9 for primary conjunctiva fibroblasts treatment were pre-determined to be 40, 25 and 20 µg/ml respectively. Primary conjunctiva fibroblast cells were seeded (200,000 per well) in a 12-well culture plate (Greiner bio-one) and cultured in serum-free DMEM/F12 medium (Gibco) for 24 hrs. Cells were washed with PBS and treated with S100A8, S100A9 and S100A8/A9 proteins in fresh media for 6 or 24 hrs. Control cells were given fresh medium without recombinant protein.

### 8. Real Time PCR

RNA was extracted with RNeasy mini kit (Qiagen) according to the manufacturer’s instructions. Reverse transcription was performed using SuperScript III first strand synthesis supermix (Invitrogen) according to manufacturer’s protocol. Real time-relative quantitative polymerase chain reaction (qPCR) was performed with SYBR Green qPCR Mastermix (Roche). Primers used for real time PCR are listed in **Table 1**. GAPDH was used as the endogenous control gene. PCR cycles were performed on the Roche LightCycler 480 with the following cycle conditions: 10 min at 95°C, 45 cycles of 10 s at 95°C, 15 s at 60°C and 20 s at 72°C, followed by melting curve analysis. The delta-delta Ct method was used for data analysis and experimental reproducibility was confirmed using 3 biological replicates. Statistical significance was evaluated at p<0.05. Pooled standard deviations of the fold changes were calculated for 3 independent experiments which used cells cultured from 3 different patients.

**Table 1:** Primers used in Real Time PCR.

Gene Symbol	Gene Name	NCBI accession number	Primer Sequence
TMSB4X	Thymosin beta 4, X-linked	NM_021109.3	F: AGACCAGACTTCGCTCGTAR: CTGCTTGCTTCTCCTGTT
CXCL1	Chemokine(C-X-C motif) ligand 1	NM_001511.3	F: CCAGACCCGCCTGCTR:CCTCCTCCCTTCTGGTCAGTT
ANXA2	Annexin A2	NM_001002857.1	F: CTCTACACCCCCAAGTGCATR: TCAGTGCTGATGCAAGTTCC
SERPINA1	Serpin peptidase inhibitor, clade A member 1	NM_000295.4	F: GTCAAGGACACCGAGGAAGAR: TATTTCATCAGCAGCACCCA
CMA1	Chymase 1	NM_001836.3	F: CTGGGGAAGAACAGGTGTGTR: TTTTGTCTTCCTGGGATTGC
GSN	Gelsolin	NM_000177.4	F: TGGTGGTGCAGAGACTCTTCCR: CTTTCATACCGATTGCTGTT
BGN	Biglycan	NM_001711.4	F: CCTTCGACCAGTCCTCCCTTCR: GGGACAGGCGAAGCCAGGTTC
VIM	Vimentin	NM_003380.3	F: CCAAACTTTTCCTCCCTGAACCR:GTGATGCTGAGAAGTTTCGTTGA
RAB10	Ras-related protein Rab-10	NM_016131.4	F: CACTTCTTCCTTGCGTCTCCR: GAAGGCATCTGGGACACATT
GADPH	Glyceraldehyde 3-phosphate dehydrogenase	NC_000012.11	F: CCCCACACACATGCACTTACCR: CCTACTCCCAGGGCTTTGATT

## Results

### 1. A Panel of Genes Found to be Dysregulated at both Transcript and Protein Level

Fold change analysis was performed using the microarray data from the 4 paired pterygium and conjunctiva tissues, a list of differentially expressed genes (fold change ≥2, p<0.05) was delineated (GEO series record number GSE51995). Some of the significantly dysregulated transcripts in pterygium are shown in **Table 2**. Other up-regulated transcripts (**Table S1**) include chemokine ligand CXCL1, biglycan (BGN), and gelsolin (GSN), annexin-A2 (ANXA2), thymosin-β4 (TMSB4X) and member of Ras oncogene family (RAB10), whereas the transcripts down-regulated in pterygium were those of Serpin peptidase inhibitor, clade A member 1(SERPINA1) and chymase (CMA1). At least 3 out of 4 pairs of the transcripts were regulated in the direction shown although p<0.05 was only for GSN and SERPINA1. We confirmed that in the dataset (http://www.ncbi.nlm.nih.gov/geo/, series accession GSE2513) from our previous study [14] these transcripts were also dysregulated in the direction shown (**Table S1**). In the previous dataset, the p values were <0.05 for 3 of these genes: BGN, GSN and TMSB4X, and these were 0.003, 0.033 and 0.014 respectively. In the current dataset, however, none of the evaluated genes, passed the Benjamini-Hochberg False discovery rate correction (p<0.05) when the paired student’s T-test was used, suggesting heterogeneity amongst different biological samples.

**Table 2:** Genes dysregulated in both transcriptomics and proteomics.

				Microarray	Proteomics
					Average iTRAQ fold change*
	Gene Symbol	Description	p	Fold change	(p<0.05)
	ALDH3A1	aldehyde dehydrogenase 3 family, member1	0.01	2.9	8.24
**Up regulated**	VIM	vimentin	0.45	1.1	4.64
	S100A8	S100 calcium binding protein A8	0.47	1.2	3.98
	S100A9	S100 calcium binding protein A9	0.64	1.2	5.93
**Down regulated**	SERPINA1	serpin peptidase inhibitor, clade A member 1	0.01	3	0.29
	TF	transferrin	0.24	1.5	0.48

*averaging obtained in duplicated analyses of 4 pairs of tissues.

The iTRAQ LC-MS/MS experimental design is shown in **Figure S1**. The complete raw data of the iTRAQ analyses are shown in **Table S2 and S3** respectively. Only proteins identified with 95% confidence were included. After relative quantification analysis, all the proteins found to be significantly different between pterygium and conjunctiva at least in 3 pairs of tissues are shown in **Table S4.** Inter-patient heterogeneity of protein expression was also observed across many proteins. When the list of dysregulated proteins in proteomics was compared against that from the microarray data, a panel of overlapping genes was generated and summarized in **Table 2**. Although not all functionally important molecules are dysregulated at both the transcript and protein levels, we decided to report and emphasize these for simplicity.

Aldehyde dehydrogenase 3 family, member1 (ALDH3A1), vimentin (VIM), S100A8 and S100A9 were found to be up-regulated in primary pterygium compared to healthy conjunctiva control. SERPINA1 and transferrin (TF) were down-regulated at both protein and transcript levels in pterygium (**Table 2**).

### 2. Validation of Over-expression of ALDH3A1 by Western Blot and Immunofluorescent Staining

**Figure 1:**
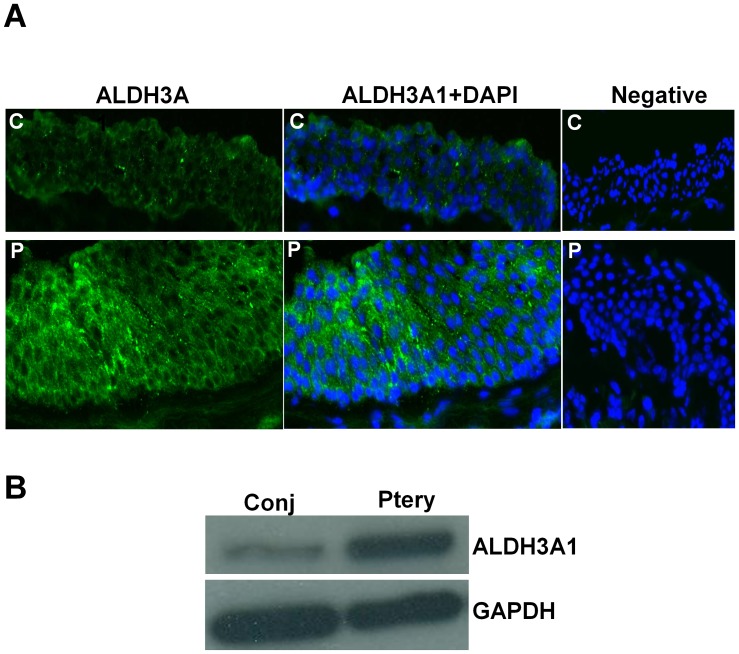
ALDH3A1 expression in pterygium. **A.** Protein expression of ALDH3A1 in both conjunctiva and pterygium fibroblast cells was determined by immunoblotting with antibodies specific for ALDH3A1 and GAPDH. **B.** Immunofluorescent staining image showing presence of ALDH3A1 (green) in human conjunctiva and pterygium epithelium. Nuclei were stained with DAPI (blue) present in the mounting medium. Negative control (without primary antibody) was shown. All images were taken at 200× magnification. C: conjunctiva; P: pterygium.

On immunofluorescent staining in pterygium and conjunctiva tissues (**Figure 1A** **,** left and middle columns), ALDH3A1 was found to be present in both conjunctival and pterygium epithelium, with a stronger expression in pterygium than in conjunctiva. The localization of the ALDH3A1 was in the cytoplasm and cell periphery of the epithelium, and present in all layers of the epithelium. Some fibroblast staining was visualized in the stroma of both pterygium and conjunctiva but comparison of staining intensity was difficult (data not shown). No fluorescent signals were detected in the negative controls (**Figure 1A** **,** right column). Levels of ALDH3A1 in primary cultured pterygium and conjunctiva fibroblast cells and epithelial cells were analyzed by western blot. Results showed that ALDH3A1 was more highly expressed in pterygium fibroblast cells than conjunctiva fibroblast cells (**Figure 1B**). However, we were not able to show similar up-regulation in cultured epithelial cells (data not shown).

### 3. S100A8, S100A9 and S100A8/S100A9 Up-regulate Disease-inducing Genes

Previously, our group has reported that S100A8 and S100A9 were localized in the superficial layer of both pterygium and normal conjunctiva epithelium, with higher levels in pterygium than uninvolved conjunctiva [21]. To test our hypothesis that S100A8/S100A9 are master regulator of many other dysregulated genes, recombinant human S100A8, S100A9 or both were used to treat conjunctiva fibroblast cells. A panel of dysregulated genes in pterygium (mentioned above) was selected as possible downstream effectors of the S100 proteins. The transcript level changes of these genes in cultured conjunctival fibroblasts, induced by addition of recombinant S100A8, S100A9 or both were evaluated by qPCR.

**Figure 2:**
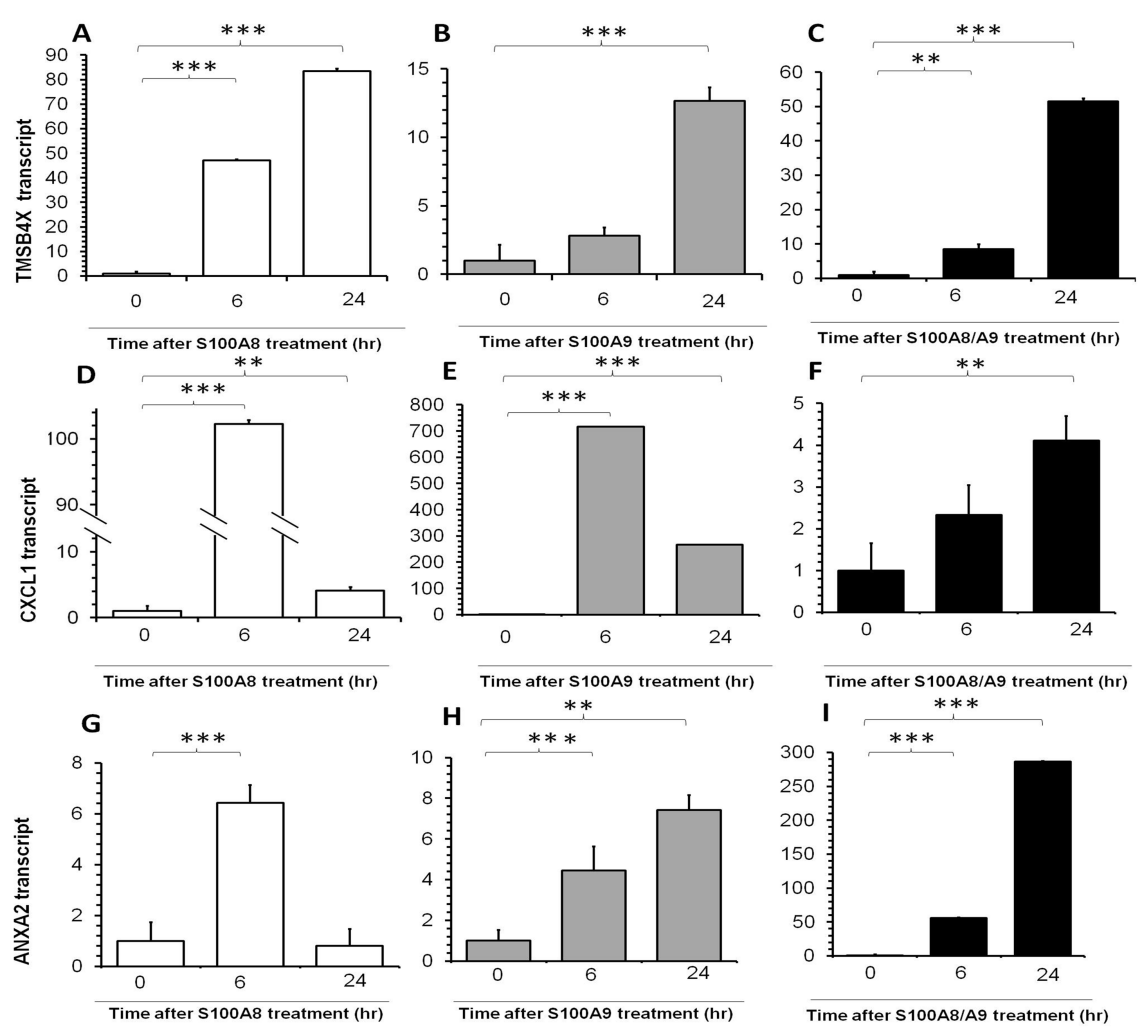
Real-time PCR quantification of fold changes in TMSB4X, CXCL1 and ANXA2 transcripts in human primary conjunctiva fibroblasts after addition of S100A8 (left column, white bars), S100A9 (middle column, grey bars) and both (right column, black bars) recombination proteins. Cells were incubated with these proteins for 6*indicates p<0.05, **indicates p<0.01 and ***p<0.001.

Inflammation-related genes TMSB4X, CXCL1 and ANXA2, were significantly increased upon addition of S100A8, S100A9 singly or in combination (**Figure 2**). TMSB4X transcript was significantly up-regulated by any of these three interventions at 6 hrs and continued to increase 24 hrs after treatment, while a significant up-regulation of TMSB4X by S100A9 was observed only at 24 hrs after treatment (**Figure 2A–C**). CXCL1 showed a marked fold change (of more than 700 folds) upon S100A8 and S100A9 treatment at 6 hrs, but this was less marked at 24 hrs. CXCL1 expression was not responsive to S100A8/A9 combination at 6 hrs but showed an increase at 24 hrs (**Figure 2D–F**). ANXA2 transcript increased significantly at 6 hrs after any of these interventions, and the increased expression persisted through 24 hrs, but this transcript was relatively more sensitive to the addition of the S100A8/A9 combination than single proteins.

**Figure 3:**
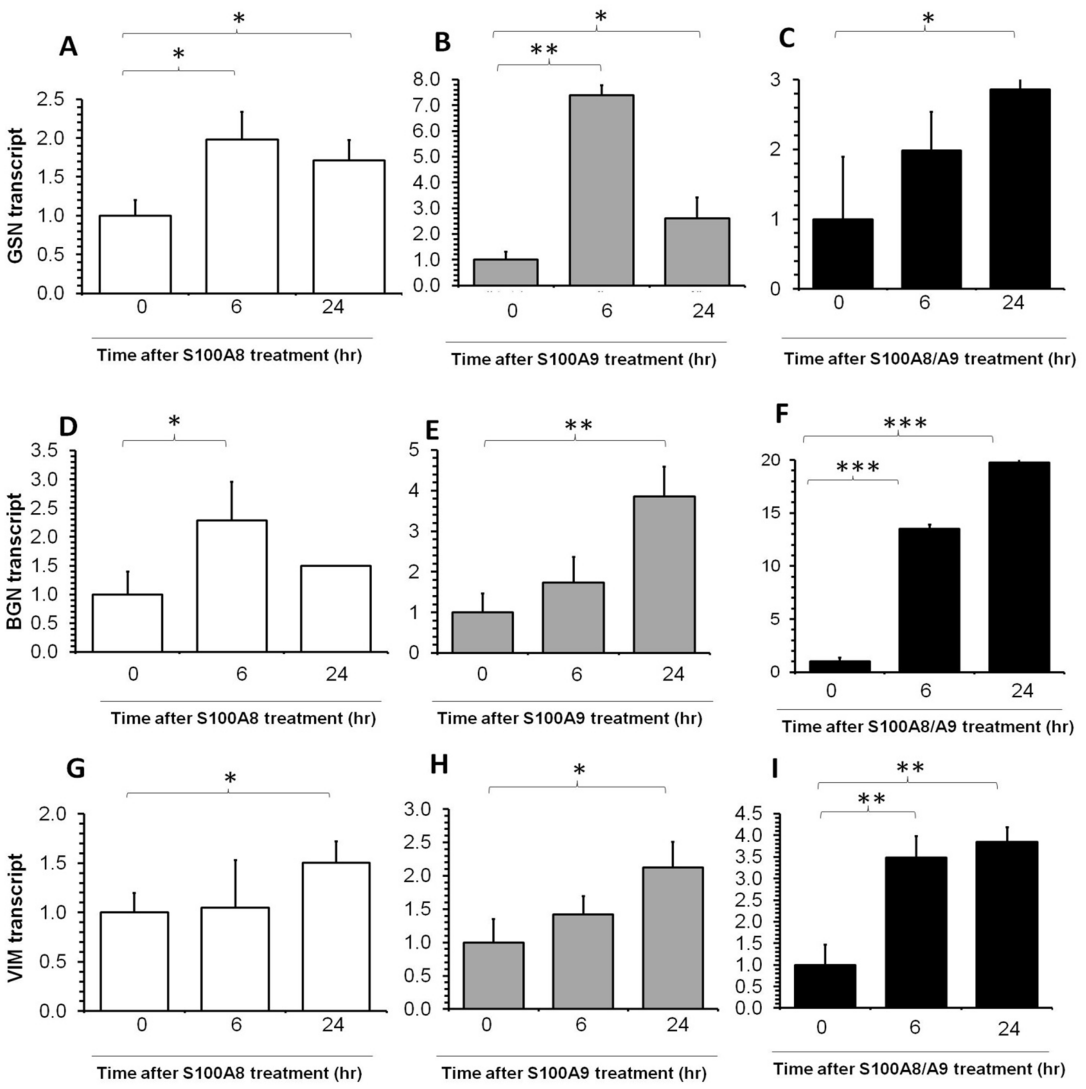
Real-time PCR quantification of GSN, BGN and VIM transcripts fold change in human conjunctiva fibroblast after S100A proteins treatment. Refer to **Figure 2** for details.

Extracellular matrix genes GSN, BGN and VIM increased upon S100A8, S100A9 or combined treatment in conjunctiva fibroblast cells (**Figure 3**), but the magnitude of change (1.5 to 20 fold) was generally not as large as the transcripts for inflammation-related genes.

**Figure 4:**
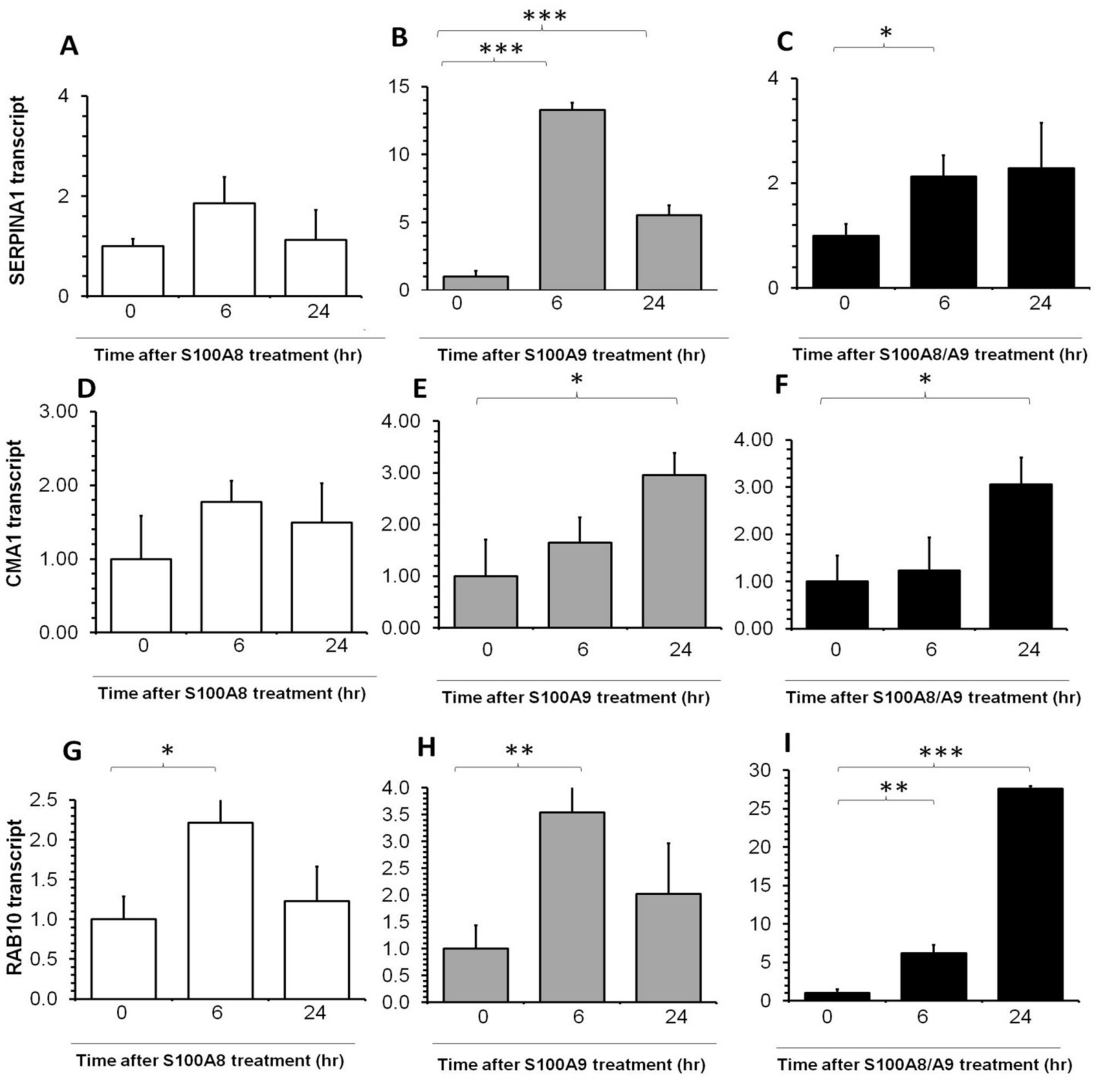
Real-time PCR quantification of SERPINA1, CMA1 and RAB10 transcripts fold change in human conjunctiva fibroblast after S100A proteins treatment. Please refer to **Figure 2** for details.

The expression levels of enzyme-coding genes SERPINA1 and CMA1 were up-regulated by S100A9 (**Figure 4B and 4E** respectively) and combination of S100A8 and S100A9 (**Figure 4C and 4F**), but not by S100A8 (**Figure 4A and 4D**). Intracellular trafficking gene RAB10 was up-regulated by S100A8 and S100A9 at 6 hrs (**Figure 4G and 4H**), while the increase persisted 6 hrs through 24 hrs for the combination treatment (**Figure 4I**).

## Discussion

In this study we reported a list of differentially expressed genes by genome-wide microarray analysis using pairs of primary pterygium and autologous conjunctiva tissues, and identified 10 dysregulated proteins by iTRAQ-LC MS/MS proteomics. Five genes were found to be dysregulated in pterygium tissue in the same directions using both approaches. In addition, we found that S100A8, S100A9 and combination could up-regulate genes related to inflammation, extracellular matrix and matrix enzymes in cultured primary human conjunctiva fibroblast cells.

A microarray study conducted by John-Aryankalayi *et al* compared two primary and one recurrent pterygium with autologous conjunctiva tissues [13]. Previously, we performed a microarray study with 4 pooled samples of conjunctiva tissues and 10 un-pooled samples of primary pterygium tissues [14]. Different with all previous studies, our current microarray utilized 4 pairs of primary pterygium and conjunctiva tissues from the same eyes. The differentially regulated genes that were further evaluated in the current study were also found to be dysregulated in the same direction in the previous dataset uploaded to GEO (http://www.ncbi.nlm.nih.gov/geo/, series accession GSE2513) [14].

To date, there have been only two published studies which identified dysregulated proteins in pterygium using proteomics [16]–[17]. Both published method utilized 2-D PAGE for protein separation and detection. While 2-D PAGE has powerful resolution, it is generally incapable of resolving proteins of extreme PI and molecular weight. Furthermore, sensitivity of the analytical system is largely dependent on the ability to visual protein spots on 2-D gels. In contrast, this study used a more modern technology involving isobaric tagging for relative and absolute quantification (iTRAQ). Coupled to LC/MS/MS, this method is capable of analyzing up to 8 samples in a single analysis thus providing a more reliable comparative analysis by eliminating the reproducibility issue encountered in 2-D PAGE. One of the 2-D PAGE based studies identified 11 proteins differentially expressed between recurrent and primary pterygium [16], but did not examine differences between primary pterygium and normal control tissue. The other study reported the over-expression of only 1 protein, peroxiredoxin 2, in primary pterygium [17].

The dysregulated genes identified were known to have biological functions relevant to inflammation or cell stress. ALDH3A1 catalyzes the oxidation of a wide variety of endogenous and exogenous aldehydes to the corresponding carboxylic acid, and is associated with cell proliferation and drug metabolism [45]. This enzyme is a corneal crystallin and plays a critical role in protecting the cornea from UV-induced oxidative stress by directly absorbing UV-radiation [46]–[48], scavenging free radicals or producing NADPH [45]. A previous study had found ALDH3A1 to be among the 25 most abundant transcripts in an un-normalized un-amplified pterygium cDNA library [13]. Over-expression of ALDH3A1 has also previously been found in tumor cells and increased ALDH3A1 expression can lead to cell proliferation [45]. The dysregulation of ALDH3A1 in pterygium tissue may be attributed to abnormal control of oxidative stress or proliferation. ALDH3A1 in cultured pterygium and conjunctiva epithelial cells showed no significant difference, most likely is related to differences in ALDH3A1 in cultured and native epithelium, the latter having matrix and fibroblast interactions.

Vimentin is a type III intermediate filament, a major cytoskeletal component of mesenchymal cells, and present in cells at the focal areas of a surgical pterygium tissue [49]. It has been reported to be involved in corneal wound healing and fibrosis [50], which are processes that may be implicated in pterygium pathology. In fact, abnormal vimentin expressing cells were reported in pterygium tissue [51].

The S100A proteins are a family of small calcium-binding dimeric proteins with pleiotropic functions, and the S100A8/A9 heterodimer play an important role in inflammatory regulation [24], [52]. S100A proteins are abundantly expressed on the ocular surface [18] and have been suggested to be involved in the pathogenesis of various ocular surface diseases, such as dry eye [20], corneal angiogenesis [19] and pterygium [20]–[21].

SERPINA1, also known as α1-proteinase inhibitor or α1-antitrypsin, is a protease inhibitor. Decreased levels of SERPINA1at both transcript and protein levels were noticed in circulating hematopoietic cells, induced by granulocyte colony-stimulating factor or chemotherapy [53]. TF is an iron-binding blood plasma glycoprotein that regulates the level of free iron in biological fluids [54].

**Figure 5:**
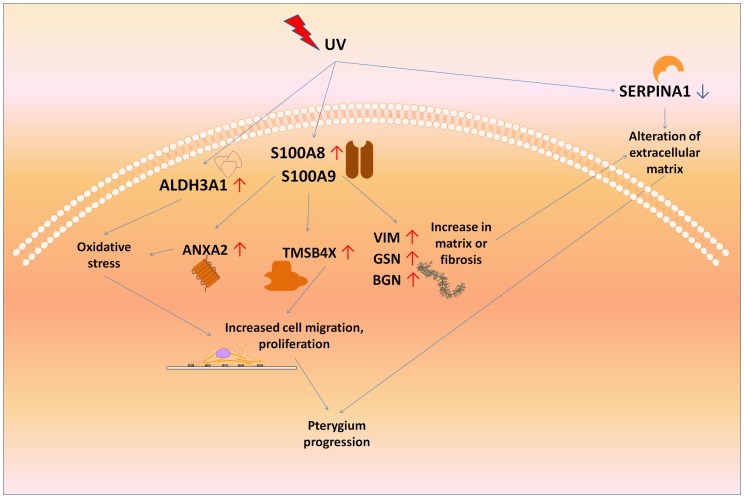
Schematic showing how UV may trigger upstream molecular disturbances such as S100A8 and S100A9, ALDH3A1, and SERPINA2 which result in a cascade of events leading to pterygium progression. ↑represents up-regulation in pterygium compared to uninvolved conjunctiva, and ↓ represents down-regulation in pterygium.

Pterygium pathogenesis is associated with increased UV exposure [1]–[4] and S100A8 protein is known to be induced by UV radiation [55]–[56]. Functions of the genes induced by addition of S100A proteins may suggest that they propagate the pterygium disease. It is possible that the S100A proteins may serve as a master regulator to activate this network of genes (**Figure 5**). In our proposed hypothesis, SERPINA1 and ALDH3A1 may also be dysregulated under the influence of UV radiation, but may not be regulated through S100A8/A9. Nevertheless, **Figure 5** shows how these pathways may be linked. For example, the ANXA2 is involved in resistance to UV-induced cell death [57]. Although we used fibroblasts in the study, other cell types such as dendritic cells, may have important responses to S100A proteins in pterygium and should be studied further.

A strength of the current study is that comparisons were done using un-pooled samples from individuals, avoiding noise related to inter-individual differences. As the amount of RNA extracted from small surgical samples of pterygium and conjunctiva was limited, earlier genome-wide gene expression studies required pooling of samples from different patients [12], [14]–[15]. Another shortcoming of sample pooling is that some of the differentially expressed genes may be obscured.

A major limitation of the protein studies is that we did not do a more definitive evaluation of small molecules like cytokines using a technique such as bead-based indirect immunofluorescent assay, as such we may not be able to verify the expression of some chemokines and cytokines. Another limitation of this study is that pterygium and conjunctiva tissues have different epithelium/stroma ratio which definitely introduce variations. This should be considered when interpreting the list of dysregulated molecules.

In conclusion, several genes were dysregulated at the transcript and protein levels in pterygium. The dysregulated molecules were consistent with previous experiments reported. The S100A8 and S100A9 have been found to be up-regulated in pterygium tissue. These may control the progression of pterygium via a network of other molecules which have also been found to be dysregulated in pterygium.

## Supporting Information

Figure S1 Four pairs of pterygium and conjunctiva samples were used for two 8-plex iTRAQ LC-MS/MS. E1 and E1 represented two 8-plex experiments. P: pterygium; C: conjunctiva. i and ii: each pair of samples was duplicated.
(TIF)

Table S1 Dysregulated gene identified by microarray.
(DOC)

Table S2 iTRAQ-LC MS/MS Results from Plate E1.
(DOC)

Table S3 iTRAQ-LC MS/MS Results from Plate E2.
(DOC)

Table S4 Dysregulated proteins identified by iTRAQ-LC MS/MS.
(DOC)

## References

[1] Di Girolamo N, Coroneo MT, Wakefield D (2003) UVB-elicited induction of MMP-1 expression in human ocular surface epithelial cells is mediated through the ERK1/2 MAPK-dependent pathway. Invest Ophthalmol Vis Sci 44: 4705–4714. 14578390 10.1167/iovs.03-0356

[2] Di Girolamo N, Kumar RK, Coroneo MT, Wakefield D (2002) UVB-mediated induction of interleukin-6 and -8 in pterygia and cultured human pterygium epithelial cells. Invest Ophthalmol Vis Sci 43: 3430–3437. 12407153

[3] Lucas RM, McMichael AJ, Armstrong BK, Smith WT (2008) Estimating the global disease burden due to ultraviolet radiation exposure. Int J Epidemiol 37: 654–667. 18276627 10.1093/ije/dyn017

[4] Nolan TM, DiGirolamo N, Sachdev NH, Hampartzoumian T, Coroneo MT, et al (2003) The role of ultraviolet irradiation and heparin-binding epidermal growth factor-like growth factor in the pathogenesis of pterygium. Am J Pathol 162: 567–574. 12547714 10.1016/S0002-9440(10)63850-3 PMC1851157

[5] Klebe S, Callahan T, Power JH (2013) Peroxiredoxin I and II in Human Eyes: Cellular Distribution and Association with Pterygium and DNA Damage. J Histochem Cytochem. 10.1369/0022155413508409 PMC3873806 24152995

[6] Perra MT, Maxia C, Corbu A, Minerba L, Demurtas P, et al (2006) Oxidative stress in pterygium: relationship between p53 and 8-hydroxydeoxyguanosine. Mol Vis 12: 1136–1142. 17093398

[7] Tsai YY, Cheng YW, Lee H, Tsai FJ, Tseng SH, et al (2005) Oxidative DNA damage in pterygium. Mol Vis 11: 71–75. 15692461

[8] Awdeh RM, DeStafeno JJ, Blackmon DM, Cummings TJ, Kim T (2008) The presence of T-lymphocyte subpopulations (CD4 and CD8) in pterygia: evaluation of the inflammatory response. Adv Ther 25: 479–487. 18483701 10.1007/s12325-008-0056-4

[9] Beden U, Irkec M, Orhan D, Orhan M (2003) The roles of T-lymphocyte subpopulations (CD4 and CD8), intercellular adhesion molecule-1 (ICAM-1), HLA-DR receptor, and mast cells in etiopathogenesis of pterygium. Ocul Immunol Inflamm 11: 115–122. 14533030 10.1076/ocii.11.2.115.15913

[10] Tsironi S, Ioachim E, Machera M, Aspiotis M, Agnantis N, et al (2002) Immunohistochemical HLA-DR antigen expression with lymphocyte subsets and proliferative activity in pterygium. In Vivo 16: 299–306. 12494867

[11] Ioachim-Velogianni E, Tsironi E, Agnantis N, Datseris G, Psilas K (1995) HLA-DR antigen expression in pterygium epithelial cells and lymphocyte subpopulations: an immunohistochemistry study. Ger J Ophthalmol 4: 123–129. 7795511

[12] Wong YW, Chew J, Yang H, Tan DT, Beuerman R (2006) Expression of insulin-like growth factor binding protein-3 in pterygium tissue. Br J Ophthalmol 90: 769–772. 16488932 10.1136/bjo.2005.087486 PMC1860207

[13] John-Aryankalayil M, Dushku N, Jaworski CJ, Cox CA, Schultz G, et al (2006) Microarray and protein analysis of human pterygium. Mol Vis 12: 55–64. 16446702

[14] Tong L, Chew J, Yang H, Ang LP, Tan DT, et al (2009) Distinct gene subsets in pterygia formation and recurrence: dissecting complex biological phenomenon using genome wide expression data. BMC Med Genomics 2: 14. 19272163 10.1186/1755-8794-2-14 PMC2670830

[15] Kuo CH, Miyazaki D, Nawata N, Tominaga T, Yamasaki A, et al (2007) Prognosis-determinant candidate genes identified by whole genome scanning in eyes with pterygia. Invest Ophthalmol Vis Sci 48: 3566–3575. 17652725 10.1167/iovs.06-1149

[16] Takashi Kanamoto NS, Ryotaro Toda, Ulfah Rimayanti, Yoshiaki Kiuch (2011) Proteomic Analyses of Proteins Differentially Expressed in Recurrent and Primary Pterygia. J Proteomics Bioinform 4: 58–61.

[17] Bautista-de Lucio VM, Lopez-Espinosa NL, Robles-Contreras A, Perez-Cano HJ, Mejia-Lopez H, et al (2013) Overexpression of peroxiredoxin 2 in pterygium. A proteomic approach. Exp Eye Res 110: 70–75. 23499777 10.1016/j.exer.2013.03.001

[18] Li J, Riau AK, Setiawan M, Mehta JS, Ti SE, et al (2011) S100A expression in normal corneal-limbal epithelial cells and ocular surface squamous cell carcinoma tissue. Mol Vis 17: 2263–2271. 21897749 PMC3164687

[19] Li C, Zhang F, Wang Y (2010) S100A proteins in the pathogenesis of experimental corneal neovascularization. Mol Vis 16: 2225–2235. 21139687 PMC2994359

[20] Zhou L, Beuerman RW, Ang LP, Chan CM, Li SF, et al (2009) Elevation of human alpha-defensins and S100 calcium-binding proteins A8 and A9 in tear fluid of patients with pterygium. Invest Ophthalmol Vis Sci 50: 2077–2086. 19168894 10.1167/iovs.08-2604

[21] Riau AK, Wong TT, Beuerman RW, Tong L (2009) Calcium-binding S100 protein expression in pterygium. Mol Vis 15: 335–342. 19223989 PMC2642841

[22] Gomes LH, Raftery MJ, Yan WX, Goyette JD, Thomas PS, et al (2013) S100A8 and S100A9-oxidant scavengers in inflammation. Free Radic Biol Med 58: 170–186. 23277148 10.1016/j.freeradbiomed.2012.12.012

[23] Sun Y, Lu Y, Engeland CG, Gordon SC, Sroussi HY (2013) The anti-oxidative, anti-inflammatory, and protective effect of S100A8 in endotoxemic mice. Mol Immunol 53: 443–449. 23127860 10.1016/j.molimm.2012.10.002 PMC3595546

[24] Lim SY, Raftery MJ, Geczy CL (2011) Oxidative modifications of DAMPs suppress inflammation: the case for S100A8 and S100A9. Antioxid Redox Signal 15: 2235–2248. 20919939 10.1089/ars.2010.3641

[25] Sroussi HY, Lu Y, Zhang QL, Villines D, Marucha PT (2010) S100A8 and S100A9 inhibit neutrophil oxidative metabolism in-vitro: involvement of adenosine metabolites. Free Radic Res 44: 389–396. 20166886 10.3109/10715760903431434 PMC2836424

[26] Sroussi HY, Kohler GA, Agabian N, Villines D, Palefsky JM (2009) Substitution of methionine 63 or 83 in S100A9 and cysteine 42 in S100A8 abrogate the antifungal activities of S100A8/A9: potential role for oxidative regulation. FEMS Immunol Med Microbiol 55: 55–61. 19087201 10.1111/j.1574-695X.2008.00498.x PMC2730662

[27] Raftery MJ, Yang Z, Valenzuela SM, Geczy CL (2001) Novel intra- and inter-molecular sulfinamide bonds in S100A8 produced by hypochlorite oxidation. J Biol Chem 276: 33393–33401. 11445563 10.1074/jbc.M101566200

[28] Harrison CA, Raftery MJ, Walsh J, Alewood P, Iismaa SE, et al (1999) Oxidation regulates the inflammatory properties of the murine S100 protein S100A8. J Biol Chem 274: 8561–8569. 10085090 10.1074/jbc.274.13.8561

[29] Vento G, Lio A, Tirone C, Aurilia C, Tana M, et al.. (2013) Association of high levels of alpha-defensins and S100A proteins with Candida mannan detection in bronchoalveolar lavage fluid of preterm neonates. Pediatr Res. 10.1038/pr.2013.60 23575874

[30] Shiotani A, Kusunoki H, Kimura Y, Ishii M, Imamura H, et al.. (2013) S100A Expression and Interleukin-10 Polymorphisms Are Associated with Ulcerative Colitis and Diarrhea Predominant Irritable Bowel Syndrome. Dig Dis Sci. 10.1007/s10620-013-2677-y 23595519

[31] Kessel C, Holzinger D, Foell D (2013) Phagocyte-derived S100 proteins in autoinflammation: putative role in pathogenesis and usefulness as biomarkers. Clin Immunol 147: 229–241. 23269200 10.1016/j.clim.2012.11.008

[32] Ehrchen JM, Sunderkotter C, Foell D, Vogl T, Roth J (2009) The endogenous Toll-like receptor 4 agonist S100A8/S100A9 (calprotectin) as innate amplifier of infection, autoimmunity, and cancer. J Leukoc Biol 86: 557–566. 19451397 10.1189/jlb.1008647

[33] Shilov IV, Seymour SL, Patel AA, Loboda A, Tang WH, et al (2007) The Paragon Algorithm, a next generation search engine that uses sequence temperature values and feature probabilities to identify peptides from tandem mass spectra. Mol Cell Proteomics 6: 1638–1655. 17533153 10.1074/mcp.T600050-MCP200

[34] Boeckmann B, Bairoch A, Apweiler R, Blatter MC, Estreicher A, et al (2003) The SWISS-PROT protein knowledgebase and its supplement TrEMBL in 2003. Nucleic Acids Res 31: 365–370. 12520024 10.1093/nar/gkg095 PMC165542

[35] Elias JE, Gygi SP (2007) Target-decoy search strategy for increased confidence in large-scale protein identifications by mass spectrometry. Nat Methods 4: 207–214. 17327847 10.1038/nmeth1019

[36] Chong PK, Lee H, Loh MC, Choong LY, Lin Q, et al (2010) Upregulation of plasma C9 protein in gastric cancer patients. Proteomics 10: 3210–3221. 20707004 10.1002/pmic.201000127 PMC3760195

[37] Chen Y, Choong LY, Lin Q, Philp R, Wong CH, et al (2007) Differential expression of novel tyrosine kinase substrates during breast cancer development. Mol Cell Proteomics 6: 2072–2087. 17855441 10.1074/mcp.M700395-MCP200

[38] Chong PK, Lee H, Zhou J, Liu SC, Loh MC, et al (2010) ITIH3 is a potential biomarker for early detection of gastric cancer. J Proteome Res 9: 3671–3679. 20515073 10.1021/pr100192h PMC3760204

[39] Ho J, Kong JW, Choong LY, Loh MC, Toy W, et al (2009) Novel breast cancer metastasis-associated proteins. J Proteome Res 8: 583–594. 19086899 10.1021/pr8007368

[40] Lim S, Choong LY, Kuan CP, Yunhao C, Lim YP (2009) Regulation of macrophage inhibitory factor (MIF) by epidermal growth factor receptor (EGFR) in the MCF10AT model of breast cancer progression. J Proteome Res 8: 4062–4076. 19530702 10.1021/pr900430n

[41] Yang Y, Lim SK, Choong LY, Lee H, Chen Y, et al (2010) Cathepsin S mediates gastric cancer cell migration and invasion via a putative network of metastasis-associated proteins. J Proteome Res 9: 4767–4778. 20812763 10.1021/pr100492x

[42] Di Girolamo N, Chui J, Coroneo MT, Wakefield D (2004) Pathogenesis of pterygia: role of cytokines, growth factors, and matrix metalloproteinases. Prog Retin Eye Res 23: 195–228. 15094131 10.1016/j.preteyeres.2004.02.002

[43] Solomon A, Grueterich M, Li DQ, Meller D, Lee SB, et al (2003) Overexpression of Insulin-like growth factor-binding protein-2 in pterygium body fibroblasts. Invest Ophthalmol Vis Sci 44: 573–580. 12556385 10.1167/iovs.01-1185

[44] Hou A, Voorhoeve PM, Lan W, Tin M, Tong L (2013) Comparison of gene expression profiles in primary and immortalized human pterygium fibroblast cells. Exp Cell Res 319: 2781–2789. 24012806 10.1016/j.yexcr.2013.08.022

[45] Muzio G, Maggiora M, Paiuzzi E, Oraldi M, Canuto RA (2012) Aldehyde dehydrogenases and cell proliferation. Free Radic Biol Med 52: 735–746. 22206977 10.1016/j.freeradbiomed.2011.11.033

[46] Estey T, Chen Y, Carpenter JF, Vasiliou V (2010) Structural and functional modifications of corneal crystallin ALDH3A1 by UVB light. PLoS One 5: e15218. 21203538 10.1371/journal.pone.0015218 PMC3006428

[47] Estey T, Cantore M, Weston PA, Carpenter JF, Petrash JM, et al (2007) Mechanisms involved in the protection of UV-induced protein inactivation by the corneal crystallin ALDH3A1. J Biol Chem 282: 4382–4392. 17158879 10.1074/jbc.M607546200

[48] Lassen N, Pappa A, Black WJ, Jester JV, Day BJ, et al (2006) Antioxidant function of corneal ALDH3A1 in cultured stromal fibroblasts. Free Radic Biol Med 41: 1459–1469. 17023273 10.1016/j.freeradbiomed.2006.08.009

[49] Kato N, Shimmura S, Kawakita T, Miyashita H, Ogawa Y, et al (2007) Beta-catenin activation and epithelial-mesenchymal transition in the pathogenesis of pterygium. Invest Ophthalmol Vis Sci 48: 1511–1517. 17389479 10.1167/iovs.06-1060

[50] Chaurasia SS, Kaur H, de Medeiros FW, Smith SD, Wilson SE (2009) Dynamics of the expression of intermediate filaments vimentin and desmin during myofibroblast differentiation after corneal injury. Exp Eye Res 89: 133–139. 19285070 10.1016/j.exer.2009.02.022 PMC2716066

[51] Dushku N, Reid TW (1994) Immunohistochemical evidence that human pterygia originate from an invasion of vimentin-expressing altered limbal epithelial basal cells. Curr Eye Res 13: 473–481. 7924411 10.3109/02713689408999878

[52] Srikrishna G (2012) S100A8 and S100A9: new insights into their roles in malignancy. J Innate Immun 4: 31–40. 21912088 10.1159/000330095 PMC3250655

[53] Winkler IG, Hendy J, Coughlin P, Horvath A, Levesque JP (2005) Serine protease inhibitors serpina1 and serpina3 are down-regulated in bone marrow during hematopoietic progenitor mobilization. J Exp Med 201: 1077–1088. 15795238 10.1084/jem.20042299 PMC2213124

[54] Neale FC (1955) The demonstration of the iron-binding globulin (transferrin) in serum and urine proteins by use of 59Fe combined with paper electrophoresis. J Clin Pathol 8: 334–337. 13271587 10.1136/jcp.8.4.334 PMC1023883

[55] Bhardwaj RS, Zotz C, Zwadlo-Klarwasser G, Roth J, Goebeler M, et al (1992) The calcium-binding proteins MRP8 and MRP14 form a membrane-associated heterodimer in a subset of monocytes/macrophages present in acute but absent in chronic inflammatory lesions. Eur J Immunol 22: 1891–1897. 1378023 10.1002/eji.1830220732

[56] Lee YM, Kim YK, Eun HC, Chung JH (2009) Changes in S100A8 expression in UV-irradiated and aged human skin in vivo. Arch Dermatol Res 301: 523–529. 19466434 10.1007/s00403-009-0960-8

[57] Yano A, Chen SP, Kita K, Jiang X, Ren Q, et al (2013) The involvement of annexin II in resistance to UVB-induced cell death and in the increased nucleotide excision repair capacity of UV-damaged DNA in human cells. Biosci Biotechnol Biochem 77: 307–311. 23391921 10.1271/bbb.120724

