# Supplementary figures and images for: Evaluation of Global Differential Gene and Protein Expression in Primary Pterygium: S100A8 and S100A9 as Possible Drivers of a Signaling Network
Source: PLoS One. 2014 May 13;9(5):e97402. doi: 10.1371/journal.pone.0097402 (PMC4019582; doi:10.1371/journal.pone.0097402)

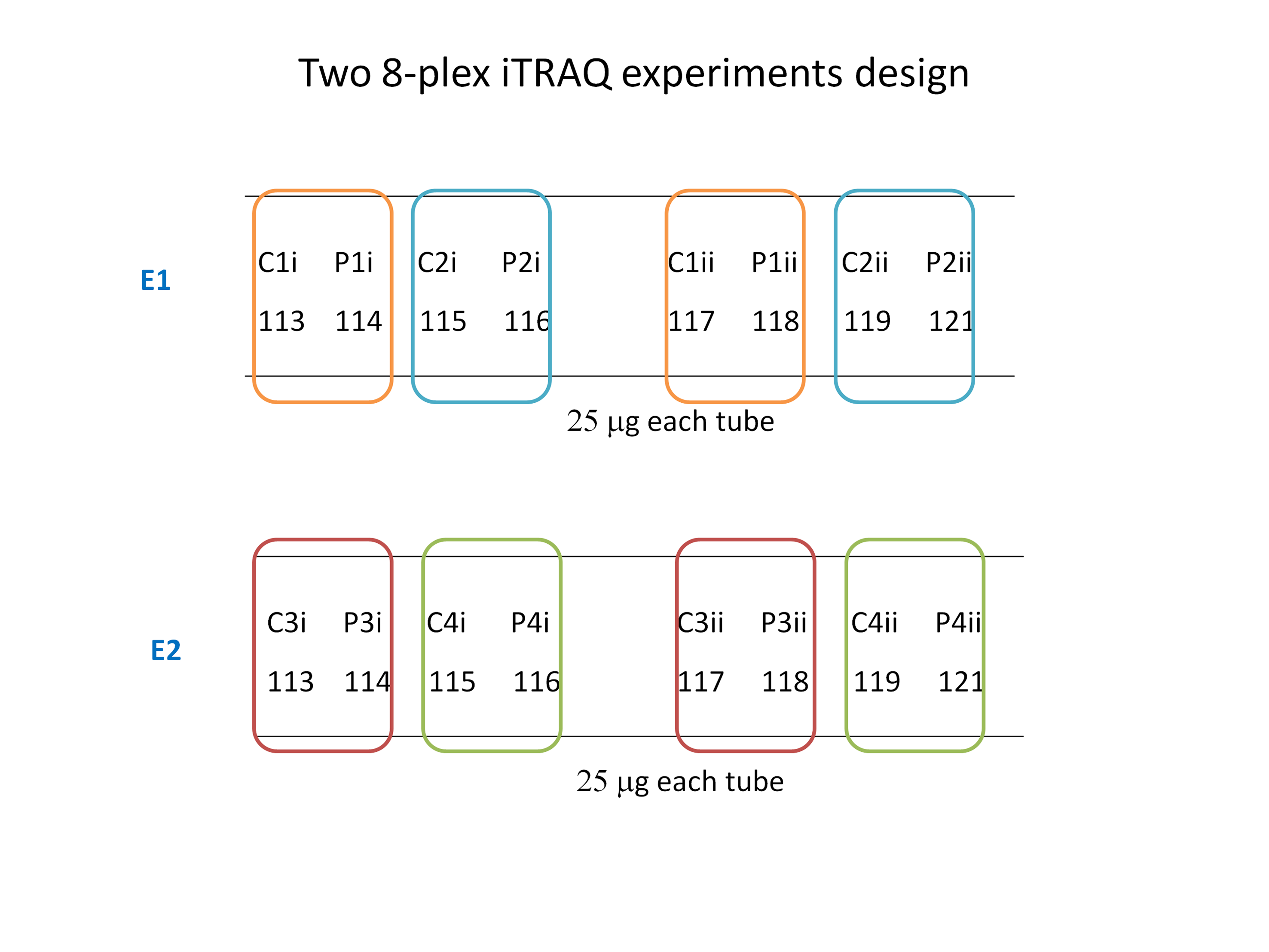

Supplement: Figure S1 — Four pairs of pterygium and conjunctiva samples were used for two 8-plex iTRAQ LC-MS/MS. E1 and E1 represented two 8-plex experiments. P: pterygium; C: conjunctiva. i and ii: each pair of samples was duplicated. (TIF) [file pone.0097402.s001.tif]
