# Supplementary material for: Evaluation of Global Differential Gene and Protein Expression in Primary Pterygium: S100A8 and S100A9 as Possible Drivers of a Signaling Network
Source: PLoS One. 2014 May 13;9(5):e97402. doi: 10.1371/journal.pone.0097402 (PMC4019582; doi:10.1371/journal.pone.0097402)
Supplement: Table S1 — Dysregulated gene identified by microarray. (DOC) [file pone.0097402.s002.doc]

**Table S1** Dysregulated gene identified by microarray

| **GenbankAccession** | **GeneSymbol** | **GeneName** | **Chromosome** | **Cytoband** | **EntrezGeneID** | **Regulation** | **PairsϮ** | **Fold Change (absolute)** |
| --- | --- | --- | --- | --- | --- | --- | --- | --- |
| NM_001511 | CXCL1 | chemokine (C-X-C motif) ligand 1 (melanoma growth stimulating activity, alpha) | chr4 | hs|4q13.3 | 2919 | up | 3/4 | 2.12 |
| NM_001711 | BGN | biglycan | chrX | hs|Xq28 | 633 | up | 4/4 | 1.74 |
| NM_198252 | GSN | gelsolin | chr9 | hs|9q33.2 | 2934 | up* | 4/4 | 1.60 |
| NM_001002857 | ANXA2 | annexin A2 | chr15 | hs|15q22.2 | 302 | up | 3/4 | 1.22 |
| NM_021109 | TMSB4X | thymosin beta 4, X-linked | chrX | hs|Xp22.2 | 7114 | up | 3/4 | 1.02 |
| NM_016131 | RAB10 | RAB10, member RAS oncogene family | chr2 | hs|2p23.3 | 10890 | up | 3/4 | 1.02 |
| NM_001836 | CMA1 | chymase 1, mast cell | chr14 | hs|14q12 | 1215 | upϮϮ | 3/4 | 1.04 |
| NM_001002236 | SERPINA1 | serpin peptidase inhibitor, clade A (alpha-1 antiproteinase, antitrypsin), member 1 | chr14 | hs|14q32.13 | 5265 | down* | 4/4 | 3.03 |

*p value (2 tail) < 0.05

Ϯ No. of pairs of pterygium/conjunctiva regulated in the direction shown in the adjacent column 'Regulation'

ϮϮ overall change is downregulated in pterygium due to the large magnitude of downregulation in 1 patient. In 3 patients, the CMA was upregulated compared to conjunctiva.
