# Supplementary material for: Evaluation of Global Differential Gene and Protein Expression in Primary Pterygium: S100A8 and S100A9 as Possible Drivers of a Signaling Network
Source: PLoS One. 2014 May 13;9(5):e97402. doi: 10.1371/journal.pone.0097402 (PMC4019582; doi:10.1371/journal.pone.0097402)
Supplement: Table S2 — iTRAQ-LC MS/MS Results from Plate E1. (DOC) [file pone.0097402.s003.doc]

**Table S2 iTRAQ-LC MS/MS Results from Plate E1**

| **Accession** | **Name** | **Peptides (95%)** | **114:113** | **PVal 114:113** | **116:115** | **PVal 116:115** | **118:117** | **PVal 118:117** | **121:119** | **PVal 121:119** |
| --- | --- | --- | --- | --- | --- | --- | --- | --- | --- | --- |
| P08670 | Vimentin | 18 | 1.63 | 0.00 | 14.06 | 0.00 | 1.67 | 0.00 | 5.60 | 0.00 |
| Q15582 | Transforming growth factor-beta-induced protein ig-h3 | 2 | 1.80 | 0.05 | 2.17 | 0.02 | 1.67 | 0.06 | 2.68 | 0.00 |
| P02743 | Serum amyloid P-component | 4 | 7.38 | 0.01 | 52.48 | 0.08 | 7.52 | 0.01 | 64.27 | 0.01 |
| P30838 | Aldehyde dehydrogenase, dimeric NADP-preferring | 14 | 18.37 | 0.00 | 1.31 | 0.15 | 21.48 | 0.03 | 0.70 | 0.47 |
| P26447 | Protein S100-A4 | 5 | 16.75 | 0.00 | 1.02 | 0.92 | 16.44 | 0.00 | 0.11 | 0.16 |
| Q06830 | Peroxiredoxin-1 | 7 | 9.91 | 0.02 | 0.84 | 0.35 | 6.73 | 0.04 | 0.16 | 0.35 |
| P31947 | 14-3-3 protein sigma | 5 | 9.29 | 0.03 | 1.69 | 0.23 | 7.59 | 0.04 | 1.63 | 0.25 |
| P62805 | Histone H4 | 10 | 7.05 | 0.00 | 0.86 | 0.77 | 11.80 | 0.00 | 0.77 | 0.34 |
| Q9BTM1 | Histone H2A.J | 5 | 6.55 | 0.01 | 0.79 | 0.54 | 5.35 | 0.01 | 0.83 | 0.57 |
| Q01469 | Fatty acid-binding protein, epidermal | 3 | 4.74 | 0.02 | 1.24 | 0.36 | 2.68 | 0.01 | 1.25 | 0.30 |
| P14618 | Pyruvate kinase isozymes M1/M2 | 6 | 4.37 | 0.00 | 1.11 | 0.66 | 2.23 | 0.02 | 0.70 | 0.52 |
| P00352 | Retinal dehydrogenase 1 | 5 | 3.98 | 0.00 | 0.63 | 0.09 | 4.33 | 0.01 | 0.35 | 0.07 |
| Q71DI3 | Histone H3.2 | 4 | 3.80 | 0.02 | 0.52 | 0.50 | 4.21 | 0.02 | 0.44 | 0.31 |
| P62937 | Peptidyl-prolyl cis-trans isomerase A | 7 | 3.40 | 0.00 | 0.84 | 0.58 | 3.50 | 0.00 | 0.79 | 0.35 |
| P04406 | Glyceraldehyde-3-phosphate dehydrogenase | 9 | 3.34 | 0.00 | 0.95 | 0.44 | 2.96 | 0.00 | 0.79 | 0.42 |
| Q99880 | Histone H2B type | 4 | 3.10 | 0.01 | 0.77 | 0.40 | 3.60 | 0.01 | 0.19 | 0.14 |
| P60709 | Actin, cytoplasmic 1 | 26 | 2.99 | 0.00 | 1.32 | 0.03 | 3.37 | 0.00 | 1.04 | 0.47 |
| P07355 | Annexin A2 | 24 | 2.96 | 0.00 | 1.14 | 0.19 | 3.37 | 0.00 | 1.11 | 0.37 |
| P06703 | Protein S100-A6 | 6 | 2.75 | 0.01 | 0.76 | 0.20 | 2.58 | 0.01 | 0.62 | 0.12 |
| P06733 | Alpha-enolase | 13 | 12.94 | 0.00 | 0.63 | 0.02 | 18.20 | 0.00 | 0.38 | 0.00 |
| P08107 | Heat shock 70 kDa protein | 12 | 8.02 | 0.01 | 0.37 | 0.01 | 13.30 | 0.00 | 0.30 | 0.00 |
| P09211 | Glutathione S-transferase P | 3 | 5.01 | 0.01 | 0.42 | 0.04 | 7.11 | 0.04 | 0.27 | 0.02 |
| P32119 | Peroxiredoxin-2 | 9 | 0.27 | 0.00 | 2.47 | 0.01 | 0.27 | 0.00 | 2.05 | 0.01 |
| P02042 | Hemoglobin subunit delta | 56 | 0.21 | 0.01 | 3.28 | 0.02 | 0.21 | 0.01 | 2.75 | 0.03 |
| P00915 | Carbonic anhydrase 1 | 5 | 0.16 | 0.01 | 4.13 | 0.04 | 0.18 | 0.01 | 4.33 | 0.02 |
| P69905 | Hemoglobin subunit alpha | 96 | 0.03 | 0.00 | 22.91 | 0.00 | 0.05 | 0.00 | 4.61 | 0.00 |
| P04083 | Annexin A1 | 10 | 6.98 | 0.00 | 0.74 | 0.09 | 15.28 | 0.00 | 0.20 | 0.01 |
| P10412 | Histone H1.4 | 4 | 2.78 | 0.00 | 0.35 | 0.00 | 2.47 | 0.01 | 0.50 | 0.06 |
| P00338 | L-lactate dehydrogenase A | 5 | 2.56 | 0.01 | 0.74 | 0.54 | 2.65 | 0.02 | 1.17 | 0.69 |
| Q09666 | Neuroblast differentiation-associated protein AHNAK | 5 | 2.61 | 0.02 | 0.55 | 0.16 | 2.51 | 0.17 | 0.11 | 0.04 |
| P31949 | Protein S100-A11 | 3 | 3.47 | 0.23 | 0.72 | 0.48 | 7.18 | 0.03 | 0.06 | 0.12 |
| P68871 | Hemoglobin subunit beta | 93 | 0.03 | 0.00 | 28.58 | 0.00 | 0.04 | 0.00 | 5.06 | 0.12 |
| P06702 | Protein S100-A9 | 11 | 0.50 | 0.42 | 2.70 | 0.00 | 1.19 | 0.87 | 1.38 | 0.01 |
| P05109 | Protein S100-A8 | 7 | 0.52 | 0.22 | 1.85 | 0.05 | 1.12 | 0.76 | 1.10 | 0.08 |
| P04004 | Vitronectin | 3 | 0.74 | 0.13 | 1.84 | 0.04 | 0.72 | 0.10 | 1.66 | 0.00 |
| P10599 | Thioredoxin | 5 | 9.29 | 0.28 | 3.66 | 0.24 | 15.14 | 0.12 | 2.49 | 0.03 |
| P04792 | Heat shock protein beta-1 | 16 | 6.92 | 0.15 | 0.44 | 0.02 | 12.82 | 0.06 | 0.45 | 0.04 |
| P51884 | Lumican | 12 | 0.49 | 0.02 | 0.68 | 0.33 | 0.49 | 0.04 | 0.59 | 0.47 |
| P01024 | Complement C3 | 5 | 0.46 | 0.01 | 1.12 | 0.62 | 0.31 | 0.00 | 0.95 | 0.83 |
| P07585 | Decorin | 7 | 0.42 | 0.01 | 0.37 | 0.36 | 0.51 | 0.01 | 0.64 | 0.48 |
| P02452 | Collagen alpha-1(I) chain | 116 | 0.38 | 0.01 | 2.96 | 0.30 | 0.42 | 0.02 | 7.52 | 0.27 |
| P02790 | Hemopexin | 2 | 0.19 | 0.01 | 0.61 | 0.26 | 0.19 | 0.01 | 0.24 | 0.31 |
| P02647 | Apolipoprotein A-I | 15 | 0.14 | 0.00 | 0.74 | 0.22 | 0.19 | 0.00 | 0.55 | 0.06 |
| P02652 | Apolipoprotein A-II | 3 | 0.13 | 0.01 | 1.02 | 0.96 | 0.29 | 0.02 | 0.34 | 0.31 |
| P01009 | Alpha-1-antitrypsin | 11 | 0.08 | 0.00 | 0.50 | 0.06 | 0.09 | 0.00 | 0.33 | 0.07 |
| P02768 | Serum albumin | 113 | 0.47 | 0.00 | 0.79 | 0.00 | 0.48 | 0.00 | 0.72 | 0.00 |
| P00738 | Haptoglobin | 8 | 0.22 | 0.00 | 0.67 | 0.06 | 0.23 | 0.00 | 0.59 | 0.05 |
| P02787 | Serotransferrin | 14 | 0.28 | 0.00 | 0.65 | 0.02 | 0.29 | 0.00 | 0.67 | 0.02 |
| P02461 | Collagen alpha-1(III) chain | 81 | 0.37 | 0.27 | 0.33 | 0.73 | 0.42 | 0.30 | 0.92 | 0.88 |
| P08123 | Collagen alpha-2(I) chain | 47 | 0.45 | 0.15 | 2.21 | 0.45 | 0.41 | 0.12 | 1.84 | 0.25 |
| P69892 | Hemoglobin subunit gamma-2 | 18 | 0.51 | 0.34 | 1.46 | 0.50 | 0.39 | 0.26 | 1.46 | 0.50 |
| P01860 | Ig gamma-3 chain C region | 10 | 0.75 | 0.61 | 0.90 | 0.84 | 0.88 | 0.81 | 0.90 | 0.86 |
| P01859 | Ig gamma-2 chain C region | 10 | 0.38 | 0.23 | 0.97 | 0.96 | 0.43 | 0.26 | 0.79 | 0.64 |
| P01857 | Ig gamma-1 chain C region | 10 | 0.26 | 0.24 | 2.56 | 0.27 | 0.28 | 0.25 | 0.22 | 0.14 |
| P02545 | Prelamin-A/C | 8 | 1.82 | 0.29 | 0.94 | 0.99 | 1.89 | 0.29 | 0.74 | 0.56 |
| P01834 | Ig kappa chain C region | 8 | 0.28 | 0.10 | 1.20 | 0.98 | 0.32 | 0.12 | 0.13 | 0.53 |
| P63104 | 14-3-3 protein zeta/delta | 7 | 0.94 | 0.61 | 0.52 | 0.39 | 0.99 | 0.68 | 0.69 | 0.53 |
| P0CG05 | Ig lambda-2 chain C regions | 6 | 0.30 | 0.13 | 0.87 | 0.40 | 0.33 | 0.18 | 0.95 | 0.93 |
| P31946 | 14-3-3 protein beta/alpha | 5 | 2.03 | 0.32 | 0.99 | 0.99 | 2.11 | 0.30 | 1.05 | 0.91 |
| Q5VTE0 | Putative elongation factor 1-alpha-like 3 | 4 | 2.19 | 0.33 | 0.84 | 0.48 | 1.87 | 0.99 | 0.82 | 0.46 |
| P08758 | Annexin A5 | 4 | 1.66 | 0.14 | 1.45 | 0.24 | 1.92 | 0.08 | 1.26 | 0.48 |
| P62258 | 14-3-3 protein epsilon | 4 | 0.70 | 0.53 | 1.08 | 0.87 | 0.72 | 0.56 | 0.01 | 0.05 |
| P62158 | Calmodulin | 3 | 2.56 | 0.25 | 1.12 | 0.82 | 2.42 | 0.26 | 0.88 | 0.81 |
| P62328 | Thymosin beta-4 | 3 | 2.13 | 0.16 | 1.96 | 0.17 | 2.15 | 0.13 | 1.13 | 0.84 |
| O43707 | Alpha-actinin-4 | 3 | 1.60 | 0.37 | 0.91 | 0.85 | 1.47 | 0.44 | 0.86 | 0.74 |
| P62987 | Ubiquitin-60S ribosomal protein L40 | 3 | 1.47 | 0.43 | 1.19 | 0.67 | 1.32 | 0.54 | 1.37 | 0.50 |
| P07437 | Tubulin beta chain | 3 | 1.34 | 0.31 | 0.88 | 0.69 | 1.29 | 0.35 | 0.50 | 0.14 |
| P67936 | Tropomyosin alpha-4 chain | 3 | 1.24 | 0.68 | 0.99 | 1.00 | 1.26 | 0.65 | 1.20 | 0.71 |
| P30043 | Flavin reductase (NADPH) | 3 | 0.65 | 0.15 | 1.18 | 0.39 | 0.59 | 0.08 | 1.39 | 0.14 |
| P02671 | Fibrinogen alpha chain | 3 | 0.59 | 0.30 | 2.19 | 0.43 | 0.54 | 0.22 | 1.69 | 0.47 |
| P01876 | Ig alpha-1 chain C region | 3 | 0.49 | 0.15 | 0.72 | 0.40 | 0.53 | 0.19 | 0.61 | 0.26 |
| P50238 | Cysteine-rich protein 1 | 2 | 3.63 | 0.18 | 0.40 | 0.29 | 3.77 | 0.18 | 0.33 | 0.24 |
| P60174 | Triosephosphate isomerase | 2 | 2.88 | 0.43 | 0.35 | 0.30 | 2.96 | 0.60 | 1.28 | 0.73 |
| P22626 | Heterogeneous nuclear ribonucleoproteins A2/B1 | 2 | 2.49 | 0.26 | 1.09 | 0.86 | 2.61 | 0.24 | 0.94 | 0.91 |
| P08727 | Keratin, type I cytoskeletal 19 | 2 | 2.27 | 0.28 | 0.48 | 0.31 | 1.57 | 0.45 | 0.52 | 0.34 |
| P15311 | Ezrin | 2 | 1.25 | 0.12 | 0.99 | 0.96 | 1.10 | 0.18 | 1.06 | 0.76 |
| P06396 | Gelsolin | 2 | 1.19 | 0.28 | 1.19 | 0.34 | 1.46 | 0.08 | 1.03 | 0.57 |
| P30044 | Peroxiredoxin-5, mitochondrial | 2 | 1.01 | 0.67 | 0.81 | 0.60 | 1.94 | 0.31 | 0.19 | 0.29 |
| P21810 | Biglycan | 2 | 0.85 | 0.76 | 2.75 | 0.23 | 0.77 | 0.64 | 2.88 | 0.22 |
| Q15661 | Tryptase alpha/beta-1 | 2 | 0.79 | 0.66 | 1.20 | 0.70 | 0.86 | 0.78 | 1.41 | 0.54 |
| P02730 | Band 3 anion transport protein | 2 | 0.69 | 0.31 | 1.01 | 0.58 | 0.77 | 0.44 | 0.87 | 0.31 |
| P01610 | Ig kappa chain V-I region WEA | 2 | 0.61 | 0.43 | 0.76 | 0.62 | 0.25 | 0.17 | 0.63 | 0.45 |
| P51888 | Prolargin | 2 | 0.59 | 0.25 | 0.84 | 0.74 | 0.52 | 0.21 | 0.77 | 0.51 |
| P15088 | Mast cell carboxypeptidase A | 2 | 0.58 | 0.39 | 1.03 | 0.94 | 0.60 | 0.42 | 0.79 | 0.66 |
| P02763 | Alpha-1-acid glycoprotein 1 | 2 | 0.39 | 0.25 | 0.75 | 0.61 | 0.38 | 0.25 | 0.37 | 0.24 |
| P23083 | Ig heavy chain V-I region V35 | 2 | 0.36 | 0.26 | 1.29 | 0.99 | 0.38 | 0.23 | 1.36 | 0.64 |
| P00918 | Carbonic anhydrase 2 | 2 | 0.28 | 0.16 | 3.25 | 0.16 | 0.30 | 0.17 | 4.21 | 0.14 |
| P01871 | Ig mu chain C region | 2 | 0.21 | 0.26 | 1.01 | 0.98 | 0.40 | 0.28 | 1.53 | 0.93 |
