# Supplementary material for: Evaluation of Global Differential Gene and Protein Expression in Primary Pterygium: S100A8 and S100A9 as Possible Drivers of a Signaling Network
Source: PLoS One. 2014 May 13;9(5):e97402. doi: 10.1371/journal.pone.0097402 (PMC4019582; doi:10.1371/journal.pone.0097402)
Supplement: Table S3 — iTRAQ-LC MS/MS Results from Plate E2. (DOC) [file pone.0097402.s004.doc]

**Table S3 iTRAQ-LC MS/MS Results from Plate E2**

| **Accession** | **Name** | **Peptides(95%)** | **114:113** | **PVal 114:113** | **116:115** | **PVal 116:115** | **118:117** | **PVal 118:117** | **121:119** | **PVal 121:119** |
| --- | --- | --- | --- | --- | --- | --- | --- | --- | --- | --- |
| P06702 | Protein S100-A9 | 13 | 9.46 | 0.00 | 6.31 | 0.00 | 11.27 | 0.00 | 4.45 | 0.00 |
| P08670 | Vimentin | 20 | 3.25 | 0.00 | 5.81 | 0.01 | 3.02 | 0.00 | 2.09 | 0.04 |
| P30838 | Aldehyde dehydrogenase, dimeric NADP-preferring | 11 | 2.29 | 0.00 | 2.27 | 0.00 | 2.81 | 0.00 | 2.21 | 0.00 |
| P05109 | Protein S100-A8 | 12 | 5.92 | 0.15 | 5.11 | 0.00 | 5.01 | 0.00 | 3.94 | 0.00 |
| P60709 | Actin, cytoplasmic 1 | 23 | 1.71 | 0.01 | 1.27 | 0.14 | 1.92 | 0.00 | 1.58 | 0.01 |
| P07355 | Annexin A2 | 19 | 1.37 | 0.21 | 2.27 | 0.01 | 1.43 | 0.20 | 2.09 | 0.00 |
| P62937 | Peptidyl-prolyl cis-trans isomerase A | 8 | 1.17 | 0.31 | 3.37 | 0.00 | 1.64 | 0.07 | 1.80 | 0.02 |
| Q99877 | Histone H2B type 1-N | 9 | 1.77 | 0.04 | 1.25 | 0.36 | 1.64 | 0.07 | 0.77 | 0.65 |
| P62805 | Histone H4 | 10 | 1.75 | 0.03 | 2.65 | 0.14 | 1.42 | 0.07 | 1.01 | 0.92 |
| Q09666 | Neuroblast differentiation-associated protein AHNAK | 4 | 1.14 | 0.43 | 1.61 | 0.11 | 1.21 | 0.41 | 2.00 | 0.02 |
| P06733 | Alpha-enolase | 16 | 0.70 | 0.03 | 0.89 | 0.25 | 0.89 | 0.23 | 1.10 | 0.19 |
| P01876 | Ig alpha-1 chain C region | 3 | 0.69 | 0.27 | 4.83 | 0.93 | 0.64 | 0.26 | 0.08 | 0.02 |
| P20774 | Mimecan | 2 | 0.62 | 0.06 | 0.61 | 0.07 | 0.58 | 0.05 | 0.51 | 0.02 |
| P02787 | Serotransferrin | 18 | 0.82 | 0.44 | 0.50 | 0.01 | 0.70 | 0.16 | 0.49 | 0.01 |
| P01024 | Complement C3 | 4 | 0.56 | 0.09 | 0.20 | 0.04 | 0.72 | 0.24 | 0.20 | 0.01 |
| P00738 | Haptoglobin | 11 | 0.42 | 0.08 | 0.34 | 0.00 | 0.42 | 0.03 | 0.22 | 0.00 |
| P32119 | Peroxiredoxin-2 | 8 | 0.29 | 0.01 | 0.54 | 0.28 | 0.32 | 0.01 | 0.33 | 0.21 |
| P00915 | Carbonic anhydrase 1 | 4 | 0.25 | 0.03 | 0.45 | 0.06 | 0.30 | 0.05 | 0.54 | 0.16 |
| P02042 | Hemoglobin subunit delta | 52 | 0.24 | 0.01 | 1.25 | 0.44 | 0.26 | 0.01 | 0.47 | 0.10 |
| P02768 | Serum albumin | 133 | 0.80 | 0.00 | 0.65 | 0.00 | 0.77 | 0.00 | 0.59 | 0.00 |
| P68871 | Hemoglobin subunit beta | 85 | 0.04 | 0.00 | 0.40 | 0.07 | 0.04 | 0.00 | 0.19 | 0.00 |
| P02647 | Apolipoprotein A-I | 11 | 0.34 | 0.00 | 0.21 | 0.01 | 0.27 | 0.00 | 0.20 | 0.00 |
| P01009 | Alpha-1-antitrypsin | 10 | 0.30 | 0.01 | 0.46 | 0.01 | 0.41 | 0.01 | 0.42 | 0.00 |
| P69905 | Hemoglobin subunit alpha | 92 | 0.05 | 0.00 | 0.33 | 0.02 | 0.04 | 0.00 | 0.11 | 0.00 |
| P02452 | Collagen alpha-1(I) chain | 96 | 1.00 | 0.73 | 0.52 | 0.26 | 1.22 | 0.60 | 0.11 | 0.20 |
| P02461 | Collagen alpha-1(III) chain | 76 | 1.13 | 0.75 | 0.72 | 0.45 | 1.60 | 0.46 | 0.60 | 0.39 |
| P08123 | Collagen alpha-2(I) chain | 34 | 1.37 | 0.37 | 0.46 | 0.29 | 2.44 | 0.10 | 0.28 | 0.26 |
| P04792 | Heat shock protein beta-1 | 13 | 0.34 | 0.69 | 1.36 | 0.21 | 0.71 | 0.45 | 1.06 | 0.87 |
| P08107 | Heat shock 70 kDa protein 1A/1B | 12 | 0.52 | 0.21 | 0.91 | 0.57 | 0.56 | 0.29 | 2.21 | 0.10 |
| P01860 | Ig gamma-3 chain C region | 11 | 1.02 | 0.96 | 0.69 | 0.52 | 1.03 | 0.95 | 0.79 | 0.66 |
| P04406 | Glyceraldehyde-3-phosphate dehydrogenase | 10 | 4.37 | 0.19 | 1.04 | 0.97 | 1.75 | 0.11 | 1.27 | 0.41 |
| P01857 | Ig gamma-1 chain C region | 10 | 0.86 | 0.78 | 0.59 | 0.41 | 0.85 | 0.76 | 0.64 | 0.46 |
| P04083 | Annexin A1 | 9 | 0.91 | 0.64 | 2.21 | 0.30 | 1.41 | 0.64 | 1.01 | 0.45 |
| P02545 | Prelamin-A/C | 9 | 1.66 | 0.25 | 1.04 | 0.72 | 1.24 | 0.56 | 0.47 | 0.26 |
| P01834 | Ig kappa chain C region | 9 | 0.68 | 0.28 | 0.47 | 0.12 | 0.92 | 0.39 | 0.03 | 0.07 |
| P01859 | Ig gamma-2 chain C region | 8 | 0.78 | 0.63 | 0.52 | 0.32 | 0.69 | 0.56 | 0.59 | 0.43 |
| P51884 | Lumican | 7 | 1.66 | 0.16 | 1.64 | 0.74 | 1.36 | 0.23 | 0.60 | 0.42 |
| Q06830 | Peroxiredoxin-1 | 7 | 1.12 | 0.46 | 1.13 | 0.46 | 1.16 | 0.36 | 1.20 | 0.29 |
| P63104 | 14-3-3 protein zeta/delta | 6 | 0.86 | 0.77 | 1.02 | 0.94 | 0.98 | 0.90 | 1.04 | 0.97 |
| P06703 | Protein S100-A6 | 5 | 1.64 | 0.60 | 1.75 | 0.35 | 1.58 | 0.48 | 1.50 | 0.57 |
| P00352 | Retinal dehydrogenase 1 | 5 | 1.61 | 0.12 | 1.79 | 0.12 | 1.29 | 0.55 | 1.08 | 0.66 |
| P14618 | Pyruvate kinase isozymes M1/M2 | 5 | 1.10 | 0.65 | 2.65 | 0.65 | 0.38 | 0.42 | 1.57 | 0.38 |
| P31949 | Protein S100-A11 | 5 | 1.08 | 0.94 | 2.27 | 0.09 | 1.06 | 0.48 | 0.07 | 0.68 |
| P07437 | Tubulin beta chain | 5 | 1.02 | 0.73 | 6.85 | 0.11 | 0.88 | 0.84 | 0.05 | 0.12 |
| P0CG06 | Ig lambda-3 chain C regions | 5 | 0.81 | 0.71 | 0.27 | 0.09 | 0.64 | 0.71 | 0.11 | 0.13 |
| P16104 | Histone H2A.x | 4 | 1.49 | 0.35 | 1.47 | 0.33 | 1.38 | 0.37 | 1.84 | 0.21 |
| P51888 | Prolargin | 4 | 1.54 | 0.12 | 1.12 | 0.58 | 1.49 | 0.21 | 0.14 | 0.18 |
| P31946 | 14-3-3 protein beta/alpha | 4 | 1.60 | 0.43 | 1.33 | 0.59 | 1.25 | 0.66 | 1.10 | 0.84 |
| P62328 | Thymosin beta-4 | 4 | 1.29 | 0.31 | 0.97 | 0.94 | 1.13 | 0.74 | 1.11 | 0.63 |
| P04075 | Fructose-bisphosphate aldolase A | 4 | 1.05 | 0.82 | 1.05 | 0.79 | 1.25 | 0.36 | 1.01 | 0.95 |
| Q5VTE0 | Putative elongation factor 1-alpha-like 3 | 4 | 1.05 | 0.66 | 0.95 | 0.83 | 0.74 | 0.22 | 1.04 | 0.90 |
| P08727 | Keratin, type I cytoskeletal 19 | 4 | 1.10 | 0.84 | 0.62 | 0.49 | 0.79 | 0.65 | 0.39 | 0.35 |
| P02763 | Alpha-1-acid glycoprotein 1 | 4 | 0.60 | 0.43 | 0.58 | 0.40 | 1.07 | 0.88 | 0.01 | 0.08 |
| P30043 | Flavin reductase (NADPH) | 4 | 0.49 | 0.25 | 0.92 | 0.25 | 0.22 | 0.08 | 0.37 | 0.25 |
| P08758 | Annexin A5 | 3 | 1.60 | 0.29 | 2.21 | 0.11 | 1.92 | 0.13 | 1.77 | 0.22 |
| Q16695 | Histone H3.1t | 3 | 1.66 | 0.16 | 1.11 | 0.59 | 1.96 | 0.10 | 2.03 | 0.32 |
| P26447 | Protein S100-A4 | 3 | 2.09 | 0.30 | 1.49 | 0.70 | 1.27 | 0.46 | 0.20 | 0.40 |
| P00338 | L-lactate dehydrogenase A chain | 3 | 1.32 | 0.25 | 1.17 | 0.57 | 1.31 | 0.24 | 0.82 | 0.46 |
| P06454 | Prothymosin alpha | 3 | 0.99 | 0.88 | 1.09 | 0.54 | 1.33 | 0.41 | 1.33 | 0.45 |
| P62158 | Calmodulin | 3 | 0.92 | 0.88 | 1.49 | 0.49 | 1.10 | 0.84 | 1.17 | 0.75 |
| P09211 | Glutathione S-transferase P | 3 | 0.65 | 0.52 | 1.32 | 0.33 | 0.93 | 0.90 | 1.38 | 0.50 |
| Q01469 | Fatty acid-binding protein, epidermal | 3 | 1.12 | 0.55 | 1.39 | 0.11 | 1.02 | 0.88 | 0.95 | 0.42 |
| P10412 | Histone H1.4 | 3 | 0.69 | 0.53 | 0.49 | 0.32 | 0.93 | 0.90 | 1.77 | 0.38 |
| P19652 | Alpha-1-acid glycoprotein 2 | 3 | 0.95 | 0.93 | 0.71 | 0.55 | 0.76 | 0.59 | 0.71 | 0.55 |
| P02790 | Hemopexin | 3 | 0.97 | 0.95 | 0.60 | 0.12 | 0.86 | 0.62 | 0.55 | 0.12 |
| P02652 | Apolipoprotein A-II | 3 | 0.79 | 0.48 | 0.38 | 0.10 | 0.73 | 0.52 | 0.51 | 0.20 |
| P07585 | Decorin | 3 | 1.56 | 0.95 | 0.55 | 0.25 | 0.65 | 0.23 | 0.33 | 0.21 |
| P00918 | Carbonic anhydrase 2 | 3 | 0.44 | 0.26 | 0.81 | 0.67 | 0.43 | 0.26 | 0.69 | 0.50 |
| P10599 | Thioredoxin | 2 | 0.79 | 0.43 | 1.54 | 0.08 | 1.29 | 0.07 | 1.43 | 0.15 |
| P19013 | Keratin, type II cytoskeletal 4 | 2 | 1.31 | 0.61 | 1.45 | 0.51 | 0.57 | 0.39 | 1.11 | 0.83 |
| P08294 | Extracellular superoxide dismutase [Cu-Zn] | 2 | 1.54 | 0.39 | 1.02 | 0.91 | 1.33 | 0.51 | 0.92 | 0.83 |
| P00558 | Phosphoglycerate kinase 1 | 2 | 1.04 | 0.83 | 1.15 | 0.55 | 1.07 | 0.73 | 1.25 | 0.38 |
| K2C5 | Keratin, type II cytoskeletal 5 | 2 | 1.08 | 0.23 | 0.82 | 0.34 | 1.04 | 0.61 | 0.65 | 0.11 |
| P30041 | Peroxiredoxin-6 | 2 | 0.77 | 0.38 | 0.96 | 0.94 | 0.77 | 0.42 | 0.80 | 0.18 |
| P60174 | Triosephosphate isomerase | 2 | 0.90 | 0.65 | 0.23 | 0.61 | 0.57 | 0.97 | 2.15 | 0.70 |
