# Supplementary material for: Evaluation of Global Differential Gene and Protein Expression in Primary Pterygium: S100A8 and S100A9 as Possible Drivers of a Signaling Network
Source: PLoS One. 2014 May 13;9(5):e97402. doi: 10.1371/journal.pone.0097402 (PMC4019582; doi:10.1371/journal.pone.0097402)
Supplement: Table S4 — Dysregulated proteins identified by iTRAQ-LC MS/MS. (DOC) [file pone.0097402.s005.doc]

**Table S4** Dysregulated proteins identified by iTRAQ-LC MS/MS

|  | **Accession** | **Gene Symbol** | **Description** | **Average iTRAQ ratio (p<0.05)** | **No. of peptides detected** |
| --- | --- | --- | --- | --- | --- |
|  | P30838 | ALDH3A1 | aldehyde dehydrogenase 3 family, member1 | 8.24 | >10 |
| **Up regulated** | P08670 | VIM | vimentin | 4.64 | >17 |
|  | P05109 | S100A8 | S100 calcium binding protein A8 | 3.98 | >6 |
|  | P06702 | S100A9 | S100 calcium binding protein A9 | 5.93 | >10 |
|  | P01009 | SERPINA1 | serpin peptidase inhibitor, clade A member 1 | 0.29 | >9 |
|  | P02787 | TF | transferrin | 0.48 | >13 |
| **Down regulated** | P02647 | APOA1 | Apolipoprotein A-I | 0.23 | >10 |
|  | P00738 | HP | Haptoglobin | 0.34 | >7 |
|  | P68871 | HBB | Hemoglobin subunit beta | 0.07 | >84 |
|  | P69905 | HBA1 | Hemoglobin subunit alpha | 0.1 | >91 |
